# Googling for Neurological Disorders: From Seeking Health-Related Information to Patient Empowerment, Advocacy, and Open, Public Self-Disclosure in the Neurology 2.0 Era

**DOI:** 10.2196/13999

**Published:** 2021-03-26

**Authors:** Mariano Martini, Nicola Luigi Bragazzi

**Affiliations:** 1 Department of Health Sciences School of Public Health University of Genoa Genoa Italy; 2 Laboratory for Industrial and Applied Mathematics Department of Mathematics and Statistics York University Toronto, ON Canada

**Keywords:** advocacy, health information seeking, neurological disorders, open self-disclosure

## Abstract

Since its introduction, the internet has played a major role in reshaping patient-physician communication and interactions, having fostered a shift from a paternalistic to a patient-centered model. Because of its dynamic nature, the internet has been used as a platform to not only disseminate knowledge—favored by improved access to an increasing wealth of available resources—but also to spread advocacy and awareness, contribute to fund-raising, and facilitate open, public self-disclosure of one’s own disease, thus eliminating any taboo and reducing the stigma associated with it. The era of Medicine 2.0 is characterized by openness, collaboration, participation, and social networking. The current situation is completely different from the time when Lorenzo Odone’s parents, after his diagnosis of adrenoleukodystrophy, decided to attend medical school in order to collect information about a devastating, unknown disease and had to contend with medical authorities at that establishment to convince them of the alleged effectiveness and safety of their discovered therapeutics. Orphan and rare neurological diseases have currently received recognition on web-based resources. However, while the intention is not to ridicule Odone’s family legacy and the “complicated lessons” they have reported, some issues should be carefully addressed by health authorities, such as the reputability, reliability, and accuracy of material available on the internet and prevention of the dissemination of material that could instill illusions and unjustified hopes in individuals seeking medical treatment. Neurologists should be aware of such digital resources, participate in web-based activities, and recommend select high-quality websites to their patients.

## Introduction

In 1984, Lorenzo Odone was diagnosed with adrenoleukodystrophy, a severe neurodegenerative disorder. His parents, Michaela and Augusto Odone, decided not to resign to unbearable and devastating pain but rather to study medicine, and—after spending much time in the libraries of the National Institutes of Health—they devised a putative treatment strategy (the so-called “Lorenzo’s oil”). Since then, 30 years have elapsed, and the situation has changed. Markedly more information is available on adrenoleukodystrophy and other rare neurological diseases; the number of voluntary health associations has notably increased, together with patient awareness; and owing to the growing availability of web-based medical material, the material that was relegated and confirmed within the university precinct and written in an obscure, technical language at the time of Odone’s family is publicly available.

Although Odone’s heritage has been undoubtedly immense, in terms of both pharmaceutical legacy and the challenge they posed to the traditional physician-patient relationship [1], ethical issues concerning the clinical effectiveness and the safety profile of new experimental therapies should be carefully considered.

Improved access to health care information has reshaped the concept of medical paternalism and has caused a shift from an “informed patient care” to a “patient-informed care” [2].

The internet and the new communication and information technologies (ICTs) have enabled physicians to deliver care remotely (the so-called “teleneurology”) [1] and have provided new tools and opportunities for disseminating and improving resident education [3]. However, undoubtedly, the major novelty lies in the profound changes associated with medical communication in the Copernican revolution: indeed, ICTs represent one of the elements of the new conceptual framework of P6 medicine, where the 6 Ps stand for “personalized,” “predictive,” “preventive,” “participatory,” “psycho-cognitive,” and “public” [4-6].

## Managing the Complexity of Neurological Disorders: Online Health Communities

Neurological diseases are particularly complex, multifactorial pathologies that require disease-specific expertise, which is rarely found in a single specialist. For neurological diseases, comprehensive management including different professionals—ranging from a neurologist to a physical therapist, occupational therapist, and speech therapist—is recommended. In this context, the concept of online health communities introduced by van der Heijk et al [7] is of particular value, specifically in the treatment of chronic diseases. ICTs can facilitate highly integrated, shared, multidisciplinary management and high-quality, affordable physician-patient interaction.

For instance, ParkinsonNet [8] represents a unique innovation developed as a network of physicians in several regions in the Netherlands, and it has numerous proven benefits and positive outcomes, including cost-effectiveness, increase in health literacy and disease knowledge, and compliance to treatment.

## Patients’ Willingness and Acceptance of the Internet and New ICTs

Patients with neurological disorders appear to actively use the internet to search for information on their pathology. They (or their relatives and parents) [9] look for resources on their prognoses, outcomes, and treatment [10] or surf the web in order find an expert specialist available for consultation [11].

One reason why internet usage is particularly widespread among patients could be linked to the fact that, because of the aging population and the increasing number of diseases and patients, physicians dedicate lesser time to them patients than before, and patients feel that their doubts and questions are not sufficiently addressed. For example, Hoch et al [12] reported that up to 20% of the users of the epilepsy “Webforum” claimed they did not receive adequate information from their physicians.

Chiò et al [13] conducted a survey among patients with amyotrophic lateral sclerosis and their caregivers and reported that approximately 55% and 83.3% of them, respectively, surfed the web for seeking health-related information. However, they were rather critical of the quality of the web-based material.

According to a survey carried out by Haase et al [14], most patients with multiple sclerosis own a personal computer and use it quite regularly. However only 20% of them would use mobile phones to communicate with their physicians, 40% would use the internet, 54% would use email, and approximately 68% would use at least one type of electronic communication device. Since the use of electronic tools was found to be a significant predictor of the acceptance of electronic interactions with health care providers, studies should particularly focus on increasing patients’ usage of new technologies and understanding which factors may act as barriers and attempting to eliminate them.

Few studies have focused on these barrier parameters. Nielsen et al [15] reported that ethnicity, vision impairment, and arm and hand disabilities markedly inhibit the use of web-based technologies in a cohort of patients with multiple sclerosis. They suggest that technological adaptation (such as voice-driven commands and text written using enlarged fonts) should be ensured in order to increase internet usage among patients.

Another potential barrier is cultural and is associated with the use of technical language, being usually adopted by medical resources. Elliott and Shneker [16] reported that only 3% of the Epilepsy Foundation’s website [17], a portal entirely devoted to seizures, adhered to the recommendations of the Institute of Medicine and the US Department of Education that health-related information should be simple and clearly written in order to be understood by any user.

## Sharing One's Own Experience and Reducing the Stigma: the Phenomenon of Open, Public Self-Disclosure

Ad hoc websites designed specifically for patients, such as PatientsLikeMe [18] or The Italian headache disorders website [19], help patients share their experiences and guide them through their difficulties in decision-making. Patients are not mere passive subjects but rather active producers and consumers at the same time (the prosumer model). Iaconesi [20] has exploited the potential of new technologies (open-source software, file uploading and sharing, and commenting and posting) to publicly self-disclose his brain cancer, thus becoming the emblematic hallmark of the new P6 medicine, reducing the associated stigma, and providing novel insights into the treatment and management of chronic neurodegenerative disorders.

## The Issue of Reliability and the Quality of the Content of Web-Based Material

An important issue associated with web-based material on neurological diseases is its quality, reputability, and reliability. Information should be written in a clear manner, providing accuracy, referenced details, and updated content. The websites should be cured in a manner that ownership, authorship, sponsorship, funding and financial support, and any other potential conflicts of interest are properly disclosed. It would be ideal for websites to be tailored to meet individual patients’ requirements, providing, for example, the opportunity to interact with experts and clinicians [21].

Peterlin et al [21] indicated high-quality and excellent websites devoted to cluster headaches, which can be important resources for patients, even though the overall quality of websites dedicated to headaches is generally poor and mediocre [22]. In total, 72.5% of these headache-related websites contained advertisements and most of them contained technical information. Efforts should be made to promote the more reputed websites, which may sometimes be difficult to come across by the general public.

Hoch et al [12] reported that only 6% of the information available on a website dedicated to epilepsy was inaccurate, whereas Di Pietro et al [23] reported that websites on neurodevelopmental disorders contained misleading information and, at least in 20% of cases, quoted scientific references in an incorrect or irrelevant manner.

Moreover, some of the online material could not be based on sufficient scientific evidence, as in the case of multiple sclerosis, for which some websites have described chronic cerebrospinal venous insufficiency (CCSVI) as the main pathogenic factor without clearly stating that this is still controversial and no scientific consensus has been reached. Instead, patients mining these websites should be advised of the fact that no controlled randomized clinical trials have been performed in order to confirm and replicate this finding. Fragoso [24] has maintained that this fraudulent ideology of describing the CCSVI theory as the “liberation treatment” could instill unjustified hopes and illusions in patients. Indeed, Bragazzi [25] reported that “CCSVI” is a highly searched term, the volume trend of which correlated with the volume trend of searching “multiple sclerosis” as a keyword.

Pucci [26] reported that the 32% of the web-based material on multiple sclerosis and Alzheimer disease was unreliable and misleading, and patients requested an intervention by a physician. Hence, it is important to conduct content analysis of web-based material. Clinicians should remain abreast of such content analyses in order to be prepared to discuss them with their patients. Furthermore, Di Pietro et al [23] suggested that “new partnerships between advocacy and experts” may ensure quality material and avoid spreading disinformation among patients.

Health authorities and organizations should establish clear standards to be followed and should monitor their adherence; this should be the onus of the medical establishment and should not be overlooked. Professionals themselves can increase their presence on web-based platforms, being active authors of weblogs or, at least, participating in the generation of websites [27].

## Positive and Negative Aspects of the New Technologies

Currently, the internet has a dual nature. It is important to emphasize that, if, on the one hand, the ICTs can positively contribute to the treatment and management of neurological disorders, on the other hand, they can have a negative impact. Regarding the positive aspects, ICTs can favor rapid, real-time dissemination of information; disseminate an unprecedented wealth of web-based relevant material; reduce stigma and discrimination; enhance social and peer support; develop new forms of physician-patient interaction and communication, which are truly patient-centered and personalized; increase health literacy; improve self-awareness and self-empowerment among patients; spread advocacy; and contribute to fund-raising. However, ICTs can also divulge potentially misleading and dangerous information regarding the etiology and management of neurological disorders, proposing ineffective and unsafe treatments. Web-based material can, indeed, be of poor quality, inaccurate, and not based on scientific evidence. Unfortunately, in the era of “fake news” and in the “post-truth age,” the risk of spreading unreliable information is quite valid [28].

## Conclusions

The internet has raised patient's awareness, facilitating the initiation and spread of self-help movements in the so called “electronic peer-to-peer virtual communities” [19,29]. It has profoundly changed the patient-physician relationship [26]; therefore, it is critical for clinicians to be fully aware of these phenomena and attempt to exploit them. For example, physicians could directly participate in developing dedicated websites or could at least have discussions with their patients about their internet usage and activities, encouraging them to properly surf the internet [26] and recommending to them a list of select high-quality websites [21,22].

Patients themselves are sometimes aware of the poor nature of the web-based material, concurrent with the findings of Marrie et al [30] that approximately 40% of patients with multiple sclerosis had concerns regarding the quality of information they obtained on the internet.

Monitoring of keywords and search hits by patients [31-39] could help clinicians understand the patients’ requirements. Predictors of internet usage and digital activities are generally associated with age, degree of symptom severity and neurological impairment, and socioeconomic status [29].

Finally, a proper understanding, the elimination of barriers to accessing information on web-based platforms, and the regulation of information—ensuring high quality standards—are important for facilitating internet usage in the era of Medicine 2.0 [40].

## Abbreviations

CCSVI: chronic cerebrospinal venous insufficiency
ICT: communication and information technology

## References

[1] Larner AJ (2011). Teleneurology: an overview of current status. Pract Neurol.

[2] Gardiner R (2008). The transition from 'informed patient' care to 'patient informed' care. Stud Health Technol Inform.

[3] Fung K, Tihan T (2009). Internet and World Wide Web-based tools for neuropathology practice and education. Brain Pathol.

[4] Bragazzi N (2013). From P0 to P6 medicine, a model of highly participatory, narrative, interactive, and "augmented" medicine: some considerations on Salvatore Iaconesi's clinical story. Patient Prefer Adherence.

[5] Bragazzi NL (2013). Children, adolescents, and young adults participatory medicine: involving them in the health care process as a strategy for facing the infertility issue. Am J Bioeth.

[6] Bragazzi NL, Del Puente G (2013). Why P6 Medicine Needs Clinical Psychology and a Trans-Cultural Approach. Health Psychol Res.

[7] van der Eijk M, Faber MJ, Aarts JW, Kremer JA, Munneke M, Bloem BR (2013). Using online health communities to deliver patient-centered care to people with chronic conditions. J Med Internet Res.

[8] ParkinsonNet.

[9] Lu C, Wirrell E, Blackman M (2005). Where do families of children with epilepsy obtain their information?. J Child Neurol.

[10] Henson JW (2008). Neurology patients online: perceptions and reality. Nat Clin Pract Neurol.

[11] Imai N, Yagi N, Konishi T, Serizawa M, Kobari M (2010). Websites offer helpful information concerning consultation with headache specialists. Cephalalgia.

[12] Hoch D, Norris D, Lester J, Marcus A (1999). Information exchange in an epilepsy forum on the World Wide Web. Seizure.

[13] Chiò A, Montuschi A, Cammarosano S, De Mercanti S, Cavallo E, Ilardi A, Ghiglione P, Mutani R, Calvo A (2008). ALS patients and caregivers communication preferences and information seeking behaviour. Eur J Neurol.

[14] Haase R, Schultheiss T, Kempcke R, Thomas K, Ziemssen T (2012). Use and acceptance of electronic communication by patients with multiple sclerosis: a multicenter questionnaire study. J Med Internet Res.

[15] Nielsen AS, Halamka JD, Kinkel RP (2012). Internet portal use in an academic multiple sclerosis center. J Am Med Inform Assoc.

[16] Elliott JO, Shneker BF (2009). A health literacy assessment of the epilepsy.com website. Seizure.

[17] epilepsy.com. The Epilepsy Foundation.

[18] PatientsLikeMe.

[19] Rossi P, Sances G, Nappi G (2005). www.cefalea.it: the first five years. J Headache Pain.

[20] Iaconesi S (2013). La Cura, An Open Source Cure for Cancer. Big Data.

[21] Peterlin BL, Gambini-Suárez E, Levin M (2007). Cluster headache: a review of online resources for patients and clinicians. Curr Pain Headache Rep.

[22] Peterlin B, Gambini-Suarez E, Lidicker J, Levin M (2008). An analysis of cluster headache information provided on internet websites. Headache.

[23] Di Pietro NC, Whiteley L, Mizgalewicz A, Illes J (2013). Treatments for neurodevelopmental disorders: evidence, advocacy, and the Internet. J Autism Dev Disord.

[24] Fragoso YD (2011). The internet racing ahead of the scientific evidence: the case of "liberation treatment" for multiple sclerosis. Arq Neuropsiquiatr.

[25] Bragazzi NL (2013). Infodemiology and infoveillance of multiple sclerosis in Italy. Mult Scler Int.

[26] Pucci E (2003). Is the internet transforming the physician-consumer relationship? Preliminary data in a neurological setting. Eur J Neurol.

[27] Lagu T, Kaufman EJ, Asch DA, Armstrong K (2008). Content of weblogs written by health professionals. J Gen Intern Med.

[28] Lavorgna L, Lanzillo R, Brescia Morra V, Abbadessa G, Tedeschi G, Bonavita S (2017). Social Media and Multiple Sclerosis in the Posttruth Age. Interact J Med Res.

[29] Shavazi M, Morowatisharifabad M, Shavazi M, Mirzaei M, Ardekani A (2016). Online Social Support for Patients with Multiple Sclerosis: A Thematic Analysis of Messages Posted to a Virtual Support Community. Int J Community Based Nurs Midwifery.

[30] Marrie RA, Salter AR, Tyry T, Fox RJ, Cutter GR (2013). Preferred sources of health information in persons with multiple sclerosis: degree of trust and information sought. J Med Internet Res.

[31] Abedi V, Mbaye M, Tsivgoulis G, Male S, Goyal N, Alexandrov AV, Zand R (2015). Internet-based information-seeking behavior for transient ischemic attack. Int J Stroke.

[32] Bragazzi NL, Bacigaluppi S, Robba C, Nardone R, Trinka E, Brigo F (2016). Infodemiology of status epilepticus: A systematic validation of the Google Trends-based search queries. Epilepsy Behav.

[33] Brigo F, Erro R (2016). Why do people google movement disorders? An infodemiological study of information seeking behaviors. Neurol Sci.

[34] Brigo F, Lochner P, Tezzon F, Nardone R (2014). Web search behavior for multiple sclerosis: An infodemiological study. Mult Scler Relat Disord.

[35] Brigo F, Otte W, Igwe S, Ausserer H, Nardone R, Tezzon F, Trinka E (2015). Information-seeking behaviour for epilepsy: an infodemiological study of searches for Wikipedia articles. Epileptic Disord.

[36] Brigo F, Trinka E (2015). Google search behavior for status epilepticus. Epilepsy Behav.

[37] Lavi-Blau T, Ekstein D, Neufeld MY, Eyal S (2016). Use of antiepileptic drugs during pregnancy and lactation: Type of information provided by searching Google. Epilepsy Behav.

[38] Milošević M, Friedrich L, Tomasović S, Bielen I (2016). Online epilepsy counseling in Croatia: What do users want to know?. Seizure.

[39] Wang H, Chen D, Yu H, Chen Y (2015). Forecasting the Incidence of Dementia and Dementia-Related Outpatient Visits With Google Trends: Evidence From Taiwan. J Med Internet Res.

[40] Eysenbach G (2008). Medicine 2.0: social networking, collaboration, participation, apomediation, and openness. J Med Internet Res.

